# RIPPLELAB: A Comprehensive Application for the Detection, Analysis and Classification of High Frequency Oscillations in Electroencephalographic Signals

**DOI:** 10.1371/journal.pone.0158276

**Published:** 2016-06-24

**Authors:** Miguel Navarrete, Catalina Alvarado-Rojas, Michel Le Van Quyen, Mario Valderrama

**Affiliations:** 1 Department of Biomedical Engineering, Universidad de los Andes, Bogotá D.C., Colombia; 2 Centre de Recherche de L’Institut du Cerveau et de la Moelle Epinière (CRICM), INSERM UMRS 975—CNRS UPR640, Hôpital de la Pitié-Salpêtrière, Paris, France; 3 Department of Electrical and Electronics Engineering, Universidad de los Andes, Bogotá D.C., Colombia; 4 Department of Electronics Engineering. Pontificia Universidad Javeriana. Bogotá D.C., Colombia; University Paris 6, FRANCE

## Abstract

High Frequency Oscillations (HFOs) in the brain have been associated with different physiological and pathological processes. In epilepsy, HFOs might reflect a mechanism of epileptic phenomena, serving as a biomarker of epileptogenesis and epileptogenicity. Despite the valuable information provided by HFOs, their correct identification is a challenging task. A comprehensive application, RIPPLELAB, was developed to facilitate the analysis of HFOs. RIPPLELAB provides a wide range of tools for HFOs manual and automatic detection and visual validation; all of them are accessible from an intuitive graphical user interface. Four methods for automated detection—as well as several options for visualization and validation of detected events—were implemented and integrated in the application. Analysis of multiple files and channels is possible, and new options can be added by users. All features and capabilities implemented in RIPPLELAB for automatic detection were tested through the analysis of simulated signals and intracranial EEG recordings from epileptic patients (n = 16; 3,471 analyzed hours). Visual validation was also tested, and detected events were classified into different categories. Unlike other available software packages for EEG analysis, RIPPLELAB uniquely provides the appropriate graphical and algorithmic environment for HFOs detection (visual and automatic) and validation, in such a way that the power of elaborated detection methods are available to a wide range of users (experts and non-experts) through the use of this application. We believe that this open-source tool will facilitate and promote the collaboration between clinical and research centers working on the HFOs field. The tool is available under public license and is accessible through a dedicated web site.

## Introduction

Studies of local high-frequency network oscillations beyond the spectral frequency limits of traditional electroencephalogram (EEG), i.e. greater than 40 Hz, have increased dramatically over the last decade. This evolution can be attributed to early animal and human studies on high frequency oscillations (HFOs) in subcortical limbic and neocortical structures, which suggest that HFOs play a role in neurological disease. For epilepsy in particular, HFOs are believed to reflect some basic neural disturbances responsible for epileptogenesis and epileptogenicity. Specifically, different studies in animals and humans have suggested that HFOs reflect abnormal synchronization due to the impairment of neuronal and network processes [1–3]. Most of these initial studies were carried out using microelectrodes and specialized systems capable of wide bandwidth recordings that could detect HFOs containing spectral frequencies up to 600 Hz [4]. More recently, the use of clinical EEG systems supporting wide bandwidths has revealed that HFOs can be also recorded using conventional depth and grid electrodes. Based on these evidences, some studies have considered the HFO density as a hallmark of the epileptic foci, which has generated a growing interest in the detection and analysis of these events [5,6].

Despite these advances, the clinical examination of such recordings remains limited mainly because the detection of these events is a challenging procedure. Specifically, HFO events have a relatively low signal to noise ratio compared to other activities (e.g., interictal epileptiform discharges). These events can occur as brief bursts lasting 30 ms or less, and they are found in brain areas capable of generating seizures [7]. In addition, the appropriate identification and analysis of HFOs requires large storage and computational capabilities [8]. This is particularly true for clinical explorations, where subjects are continuously monitored for days, weeks or even months, creating massive amounts of data to be stored and processed. This data is usually acquired at high sampling rates, varying from 10–30 kHz, 1–4 kHz or below 1 kHz for intracranial micro and macro electrodes, or surface EEG respectively, where several locations in the brain are simultaneously acquired (varying from dozens to hundreds of contacts). To complement this, there is not a general criterion for the identification of HFO events, and their demarcation differs between experts [9]. Indeed, during the HFO validation process, the inter-rater reliability should be taken into account [10], but currently there is not a common procedure to share HFO analyses across different groups.

To overcome all these issues, the following two strategies have been proposed: the development of analytic techniques that can reliably detect HFOs in continuous wide bandwidth EEGs recorded from microelectrodes and conventional clinical electrodes and the creation of infrastructure projects that facilitate the sharing of wide bandwidth data [9]. Specifically in terms of the first strategy, several methods for the automatic detection of HFOs have been recently developed [11–18]. Nevertheless, despite efforts to develop a reliable procedure to correctly identify HFOs, there is currently no consensus about the one method that performs best in all contexts. As solution to this, it has been suggested that different algorithms for HFO detection should be tested across similar datasets to improve the performance of automatic HFO detection methods, and these algorithms should permit the change of multiple parameters on the go if meticulous studies are required, [9,14]. It is important to consider that most of the developed methods require sophisticated mathematical and computational tools for their implementation and operation, which can substantially reduce the detection algorithms utility in contexts where non-technical researchers and clinicians could potentially benefit. Also, within these strategies, it is required the development of analysis tools that can accurately characterize and quantify this type of events including the possibility to share the HFO analyses. Though several open-source computational tools exist for the analysis of neural data (see for instance MEA-Tools [19], EEGLAB [20]; SigTOOL [21]; BioSig [22]; FieldTrip [23]; AnyWave [24]), none of them have been designed for the analysis of HFOs, and they lack the adequate environment for manual and automatic detection and validation of this type of events as well as the implementation of several HFOs detection algorithms. Consequently, some groups have developed customized interfaces to test their own algorithms [25,26], or they have joined professional EEG companies to develop proprietary software [27]. Unfortunately, these applications currently remain insufficiently documented or non-available to the general public, restricting their use and validation and making difficult the further support for comprehensive HFO analyses.

To achieve a practical and reliable tool for HFO analysis, we developed RIPPLELAB, a MATLAB open-source application (Mathworks®, Natick, MA, USA). This tool integrates several methods for HFO detection within an easy-to-use graphical user interface (GUI), which assists clinicians and researchers in the identification, selection and validation of HFOs. In particular, RIPPLELAB allows users to implement different quantitative measures to optimally identify and classify HFOs, reducing significantly the time required to visualize and validate putative events. RIPPLELAB also proposes a common procedure to share HFO analyses in order to promote collaborations across research centers. This tool was designed to be manipulated by researchers and clinicians with no programming skills, and users do not require any programming abilities to execute an analysis. More seasoned scientists can expand the possibilities through the configuration of advanced parameters or the inclusion of complementary modules

## Materials and Methods

### HFOs Detection Methods

HFOs are cortical discharges observed in the EEG. They are usually defined as local field potentials (LFP) of short duration (30–100 ms), with more than three oscillations distinguishable from the background activity [28]. HFOs are usually categorized depending on their main frequency component as *gamma* (60–150 Hz), *ripples* (150–250 Hz) and *fast ripples* (> 250 Hz), where gamma are essentially considered physiological oscillations, and fast ripples are mainly studied as pathological oscillations [29,30]. Conversely, ripples can be considered physiological or pathological oscillations according to different factors such as their recording region [31,32]. In particular, pathologic epileptic HFOs can occur as independent events or together with spikes [5,9]. Detection methods exploit all these characteristics to identify HFOs from background.

Broadly speaking, HFO detection methods can be classified in three groups: manual review, supervised detection and unsupervised detection. Manual review is performed by visual inspection of an expert with required knowledge on electrophysiological recording and signal processing to distinguish putative events from filtered artifacts [33]. Supervised detection relies on detection methods with high sensitivity and low specificity detection, which is complemented with manual review. Finally, unsupervised detection requires methods with high sensitivity and high specificity to be effective, which is difficult to achieve in databases with dissimilar characteristics [4,9].

HFO detection by manual review is performed by visual inspection of EEG recordings in parallel with a frequency representation of the same recording. Two methods of visual marking are commonly used: the visual inspection of the time-frequency plot in parallel with the focus electrodes [34], and the simultaneous inspection of raw data, filtered data > 80 Hz and filtered data > 250 Hz [28]. By definition, two main issues arise when the visual inspection methodology for HFOs detection is applied: (i) a high subjectivity because the visual marking is influenced by the perception of the reviewer, and (ii) the increased time-consuming of the process that limits the amount of signal to be analyzed by the reviewer, which is usually in the interval between 5–10 minutes [10]. Yet, this method is considered the gold standard when assessing the performance for most of the automated algorithms for HFO detection [35].

In general, the supervised and unsupervised detection algorithms achieve a series of common steps for detection of putative events. The first step is to emphasize the frequency of interest by filtering the raw signal [4]. Then, a detection measure for thresholding is implemented, which can be based on energy, statistical or spectral characteristics of the filtered data. Finally, depending on the method, a supervised or unsupervised mechanism for discriminating HFOs from noise, artifacts and spikes is implemented.

The HFO detection methods have been developed according to the needs of each research center: The first supervised method for the automatic detection of HFOs was implemented by Staba et al. [11]. This method analyzes 10-min EEG segments in frequencies between the 80 and 500 Hz by applying a band-pass filter, and subsequently a root mean square (RMS) for the detection of HFO events. The authors reported a sensibility of 84%. Gardner et al. [13] implemented a similar strategy by selecting putative HFOs through the evaluation of the *Line Length* energy from the equalized filtered signal in 3-min epochs. The reported sensitivity was 89.5%. Crèpon et al. [12] applied the Hilbert envelope to select putative events, achieving a reported sensitivity of 100% and a specificity of 90.5%. The first unsupervised method for HFO detection to our knowledge was developed by Blanco et al. [15]. The authors processed 10-min EEG signal identifying putative HFOs using the Staba’s method, and a series of features from each event were computed to develop a K-Medoids/Gap-Statistic clustering to separate different types of events, where true and false HFOs were characterized. In this study, the authors reported no measures of sensitivity or specificity. Zelmann et al. [14] implemented a supervised method to improve HFO detection in EEG signals with continuous high frequency activity. They reported a sensitivity and specificity of 91%. Dümpelmann et al. [36] developed another unsupervised method adjusted to the detection of ripples. This method selects the RMS amplitude, the *Line Length* energy and the instantaneous frequency as main features, and then a radial basis function neural network is used to select real events. In this work, the authors reported a sensitivity of 49% and specificity of 36.3%. Another supervised algorithm was implemented by Birot et al. [26] to detect fast ripples in the 250–600 Hz band. This algorithm uses the RMS amplitudes and a Fourier or Wavelet energy ratio to detect putative events. The authors reported a sensitivity of 93% and a specificity of 95% with optimal parameters. Matsumoto et al. [3] applied a support vector machine with features extracted from putative HFOs recognized by a *Line Length* detection, employing spectral characteristics for training. The authors reported a sensitivity of 83.8% and a specificity of 84.6%. Burnos et al. [17] implemented a Hilbert envelop detection and they included a time-frequency analysis for a spectral characterization of the selected events; the authors reported a total sensitivity of 50% and specificity of 91%. Finally, a recent unsupervised algorithm was developed by Gliske et al. [18], where a parallel detection of artifacts and events is implemented. This study reports a sensitivity of 78.5% and specificity of 88.5%.

#### Algorithms for automatic detection of HFOs

Four methods for automated detection were implemented in RIPPLELAB. They were selected based on the following three characteristics: (i) the detection method is supervised, (ii) the algorithm has the ability to detect ripples and fast ripples, and (iii) the sensitivity reported is higher than 80%. Nonetheless, because of the RIPPLELAB’s modular structure, other methods for HFO detection can be easily integrated into the application if desired.

**Fig 1:**
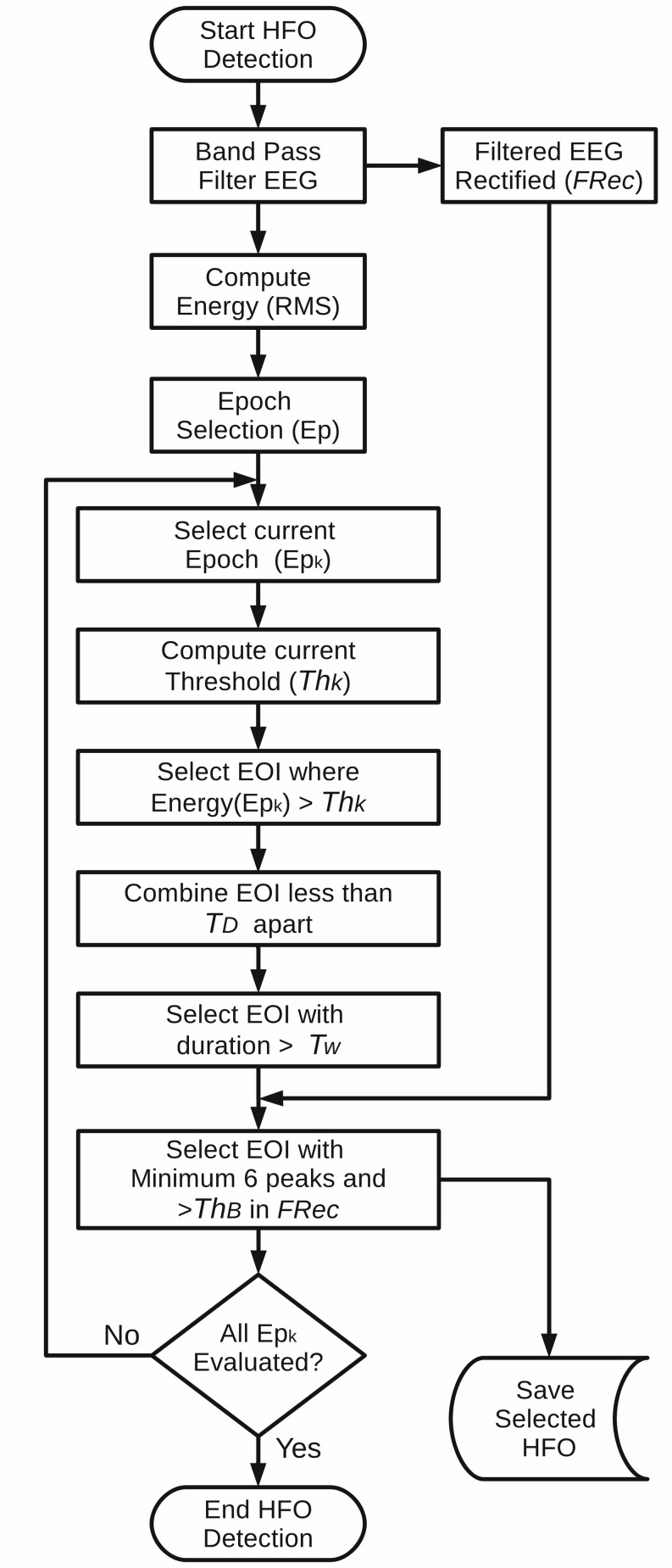
Algorithm flowchart for the implemented STE detection method. The epoch analysis is included in order to analyze long-term recordings computing the energy threshold (Th_k_) with local energy. In Staba et al. [11] the parameters are set as follows: Th_k_ = 5-SD, T_D_ = 10ms, T_w_ = 6ms and Th_B_ = 3-SD. Epoch (Ep_k_) Time = 600s. *EOI*: Events of interest.

The first implemented method, Short Time Energy (STE) is the algorithm proposed by Staba et al. [11]. In brief, the wideband EEG signal is band-pass filtered in the high frequency range. The energy from the filtered signal is then computed using the RMS defined by the equation

$$E\left(t\right)=\sqrt{\frac{1}{N}\;\sum_{k=t-N+1}^{i}x^{2}\left(k\right)}$$ (1)

within a N = 3 ms window, and successive RMS values greater than 5 standard deviations (SD) above the overall RMS mean are selected as putative HFO events if they last more than 6 ms. Finally, only the events containing more than 6 peaks greater than 3 SD above the mean value of the rectified band-pass signal are retained. In addition, the events separated by 10 ms or less are marked as a single oscillation. In the original paper, the estimation of the energy threshold depended on the complete analyzed segment, which had a duration of 10-min. In RIPPLELAB, the energy threshold can be computed for the entire signal, as originally proposed by Staba et al. [11], or for shorter segments, as suggested by Gardner et al. [13]. The flowchart of the implemented STE algorithm is shown in **Fig 1**.

**Fig 2:**
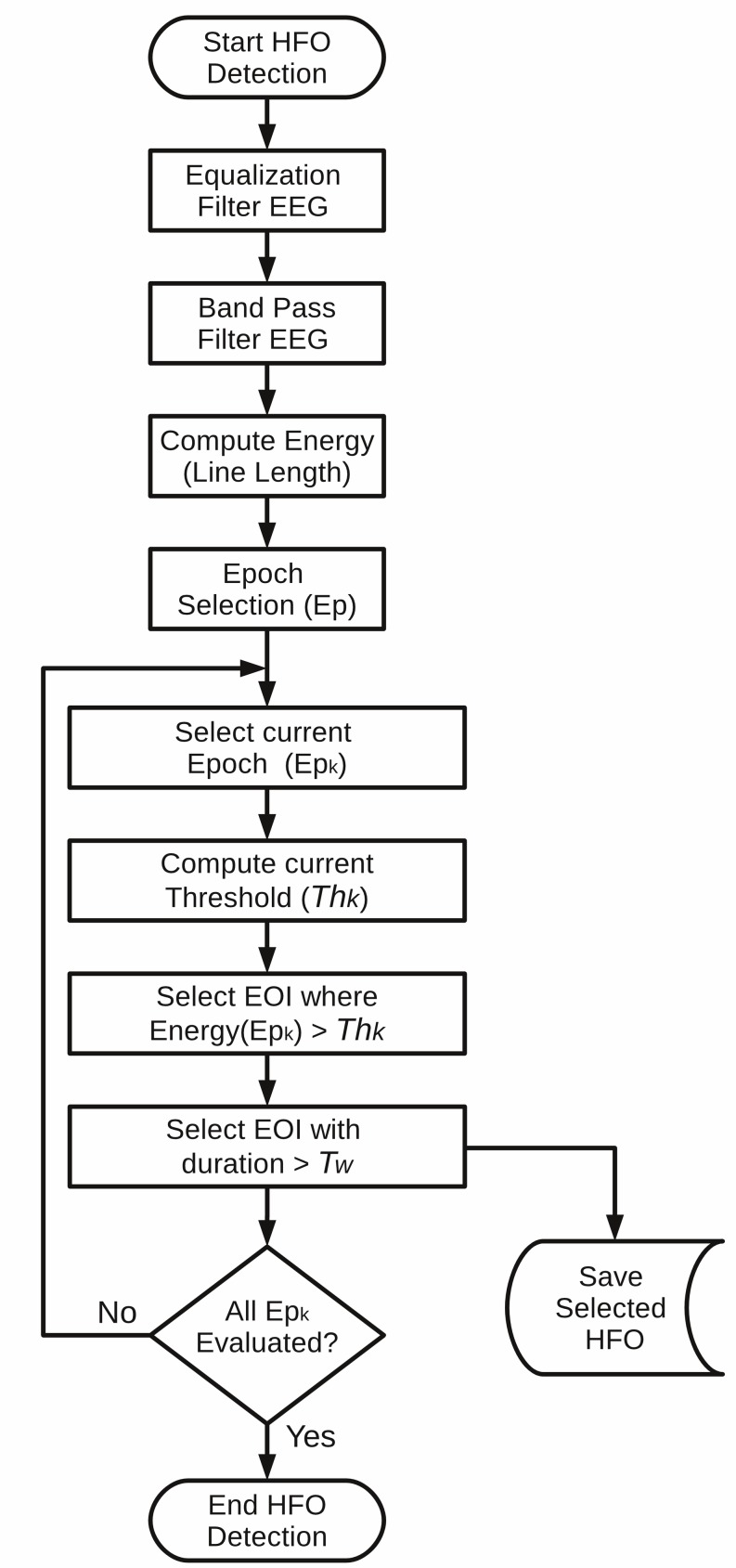
Algorithm flowchart for the implemented SLL detection method. In Gardner et al. [13] the parameter Th_k_ is set as the 97.5 percentile of the energy epoch (Ep_k_); we set as default T_w_ = 12 ms to include events larger than 6 oscillations at 500Hz. Epoch (Ep_k_) Time = 180s. *EOI*: Events of interest.

The second implemented algorithm named Short Line Length detector (SLL), was developed by Gardner et al. [13,31]. In this approach, a preprocessing stage is done with a derivative filter in order to equalize the spectrum of the signal. Next, a band-pass filter is applied. From this, the energy of the signal is calculated by a short time line length measure [37] defined by

$$E\left(t\right)=\sum_{k=t-N+2}^{i}\left|x\left(k\right)-x\left(k-1\right)\right|$$ (2)

with window N = 5 ms. An event is valid if its amplitude is greater than the 97.5th percentile of the empirical cumulative distribution function for each 3-min epoch and if it has a minimum duration. This duration is set to 80 ms in [13], but it is ignored in [31]. We set this parameter to 12 ms by default in order to accept events larger than 6 oscillations at 500 Hz. The flowchart of the implemented SLL algorithm is shown in **Fig 2**.

**Fig 3:**
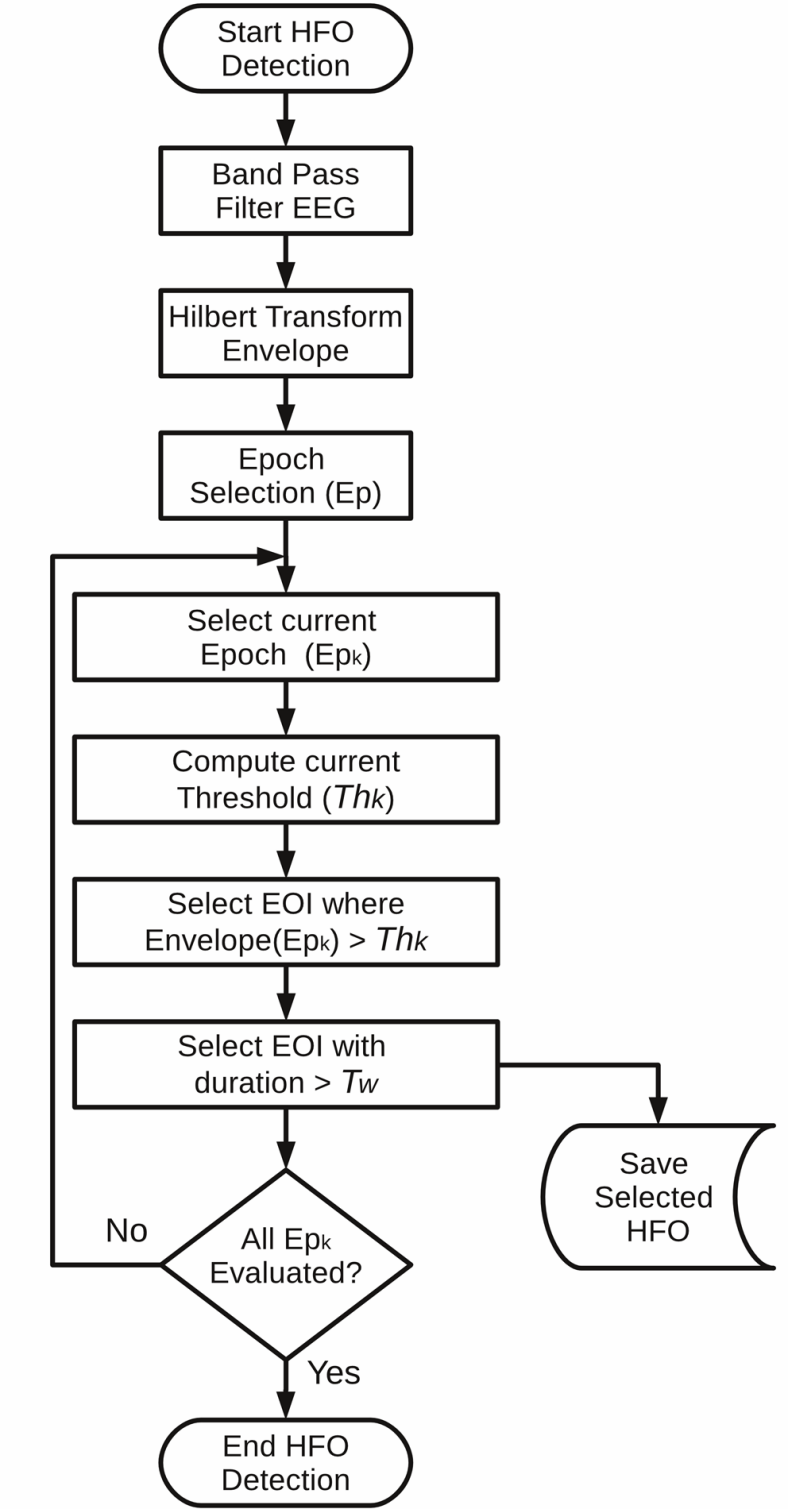
Algorithm flowchart of the implemented HIL detection method. The epoch analysis is included in order to evaluate long-term recordings computing the envelope threshold (Th_k_) with local variations of amplitude. As proposed by Crèpon et al. [12], the parameters by default are: Th_k_ = 5-SD and T_w_ = 10 ms. Epoch (Ep_k_) Time = 3600s. *EOI*: Events of interest.

The third method we included was proposed by Crépon et al. [12]. In this method, the signal is first filtered between a selected frequency range, and the envelope is then computed with the Hilbert transform. For an event to be considered valid, two conditions must be met: first, for each event, the local maximum must exceed a threshold of 5 SD of the envelope calculated originally over the entire recording or from a time interval. Second, each detected HFO must have a minimal time length of 10 ms. This method is called Hilbert Detector (HIL) in RIPPLELAB, and its flowchart is presented in **Fig 3**. As in the STE detector case, we included the possibility to analyze the threshold by epochs specified by the user.

**Fig 4:**
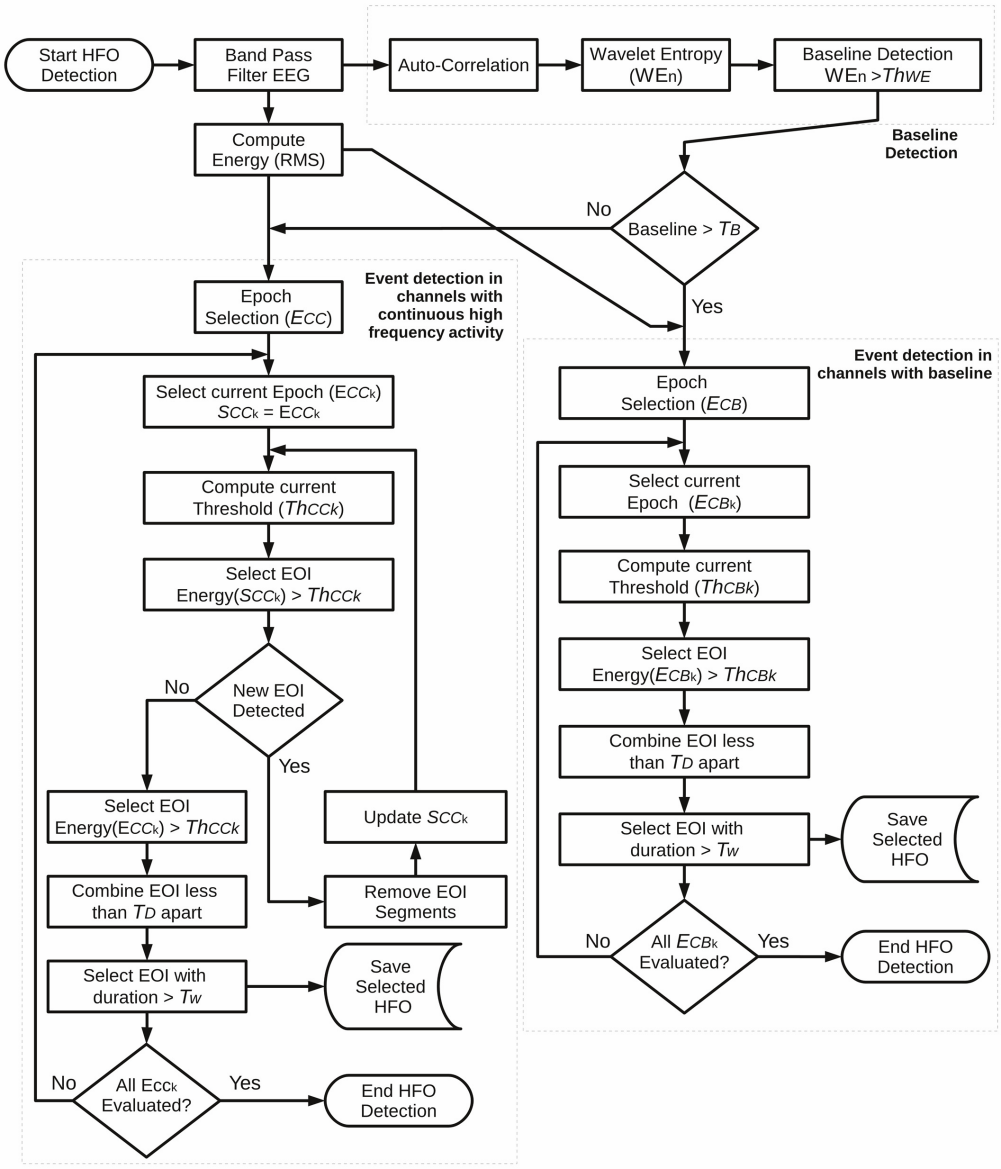
Algorithm flowchart of the implemented MNI detection method. This method is implemented according to the following two detection steps: (i) A baseline detection based on the wavelet entropy (WE_n_) over an entropy threshold (Th_WE_), and (ii) the HFO detection, which depends on the quantity of detected baseline. If the quantity of detected baseline is greater than the threshold T_B_, then the HFO detection is processed by selecting events with energy higher than Th_CBk_ in each epoch (E_CB_). If the baseline is not enough, then an iterative process is carried out in order to find an energy threshold (Th_CCk_) that detects the highest quantity of putative events. Events are selected if they have a duration greater than T_w_. As published by Zelmann et al. [14], the parameters by default are set as Th_WE_ = 0.67, T_B_ = 5s/min, Th_CBk_ = 99.9999 percentile, Th_CCk_ = 95 percentile, T_D_ = 10 ms, T_w_ = 10 ms, E_CB_ time = 10s, E_CC_ time = 60s. *EOI*: Events of interest.

The last algorithm incorporated, the MNI detector (MNI) was developed by Zelmann et al. [14]. In this method the signal is first band-pass filtered. Then, a baseline detection procedure based on the wavelet entropy is applied [38]. For this, the signal is divided into segments of 125 ms with 50% overlap. Next, for each segment, the normalized wavelet power of the autocorrelation function is computed using the complex Morlet wavelet [39]. Subsequently, the maximum theoretical wavelet entropy from the segment is obtained for the white noise [40], and the segment is considered as a baseline interval when the minimum entropy is larger than a threshold. If a sufficient amount of baseline exists, HFO candidates are detected in accordance with the energy, defined as the moving average of the RMS amplitude of the filtered signal. Segments with energy above a threshold and lasting more than 10 ms are considered as HFOs. Similar to other methods, events located less than 10 ms apart are considered as single events. If a sufficient amount of baseline is not present in the signal, an iterative procedure is carried out where the threshold is computed for the band-passed signal. Originally, this detection methodology was implemented with 1-min segments of EEG signal. In addition to this, we included the possibility to process the data thresholds in epochs of time specified by the user. The flowchart of the MNI algorithm is presented in **Fig 4**.

### Software Overview

**Fig 5:**
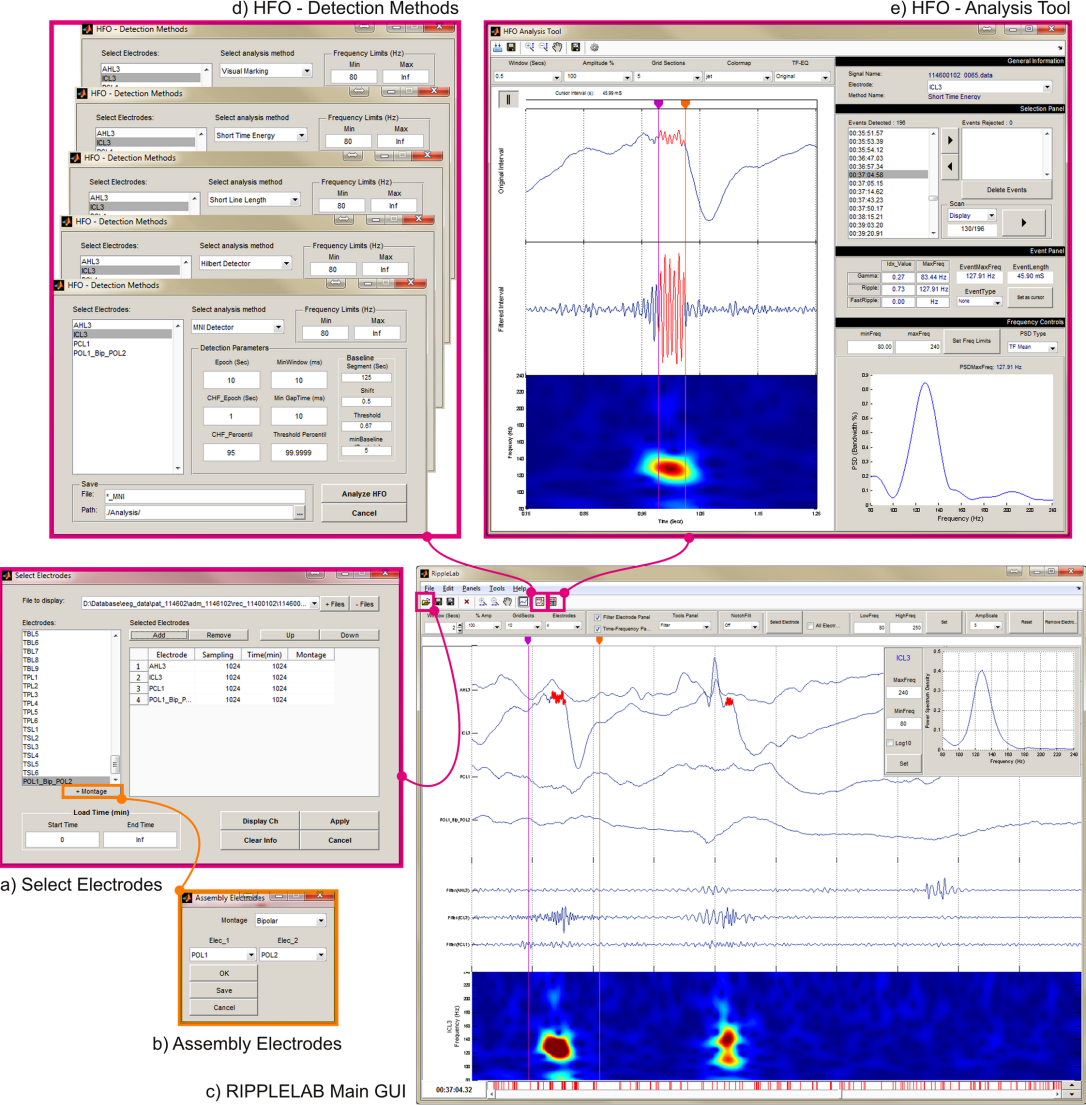
RIPPLELAB multi-window approach. The user interface is constructed as an intuitive tool for HFO analysis. A typical analysis can be carried out as follows: (a) In the *Select Electrodes* window, the user selects the files and electrodes to analyze. The user has the option to display the electrodes selecting the *Display Ch* Button or just to save the selection without plotting electrodes with the *Apply* button. (b) If bipolar or average montages are needed, the *Assembly Electrodes* window gives options for this purpose. (c) If the user chooses to draw the electrodes, controls for handling the display are available in the main GUI. Here, the user has the possibility to inspect further information from the displayed signals with tools such as filters, frequency spectrum, time-frequency plots and time cursors. (d) In the *HFO–Detection Methods* window the parameters for HFO detection are set and the detection method is launched. (e) In the *HFO Analysis Tool* window, the detected events are validated. Several options have been included in order to provide different criteria for the validation of real HFOs.

RIPPLELAB is a multi-window GUI developed in MATLAB for the analysis of high frequency oscillations. It is intended to be a user-friendly and intuitive tool, where users with technical and non-technical backgrounds can explore and analyze brain oscillations from different types of electrophysiological data, especially at high frequency ranges. RIPPLELAB has been released under GNU Public License version 3, and the source code and documentation can be found in https://github.com/BSP-Uniandes/RIPPLELAB/. The code was originally written in MATLAB version 7.12, and it is compatible with later versions. The tool was developed with a modular design, allowing expert users to modify and integrate new developments. RIPPLELAB was developed exclusively using MATLAB scripts; therefore, the compilation of native libraries or external functions is not required. This multi-platform tool can be used on OS X, Linux and Windows 32 and 64-bit architectures, and it can be installed as a MATLAB App in versions equal or later to R2012b. The RIPPLELAB multi-window approach is displayed in **Fig 5**. We chose MATLAB as programming platform because of its widely extended usage in the epilepsy and HFOs research environment.

**Fig 6:**
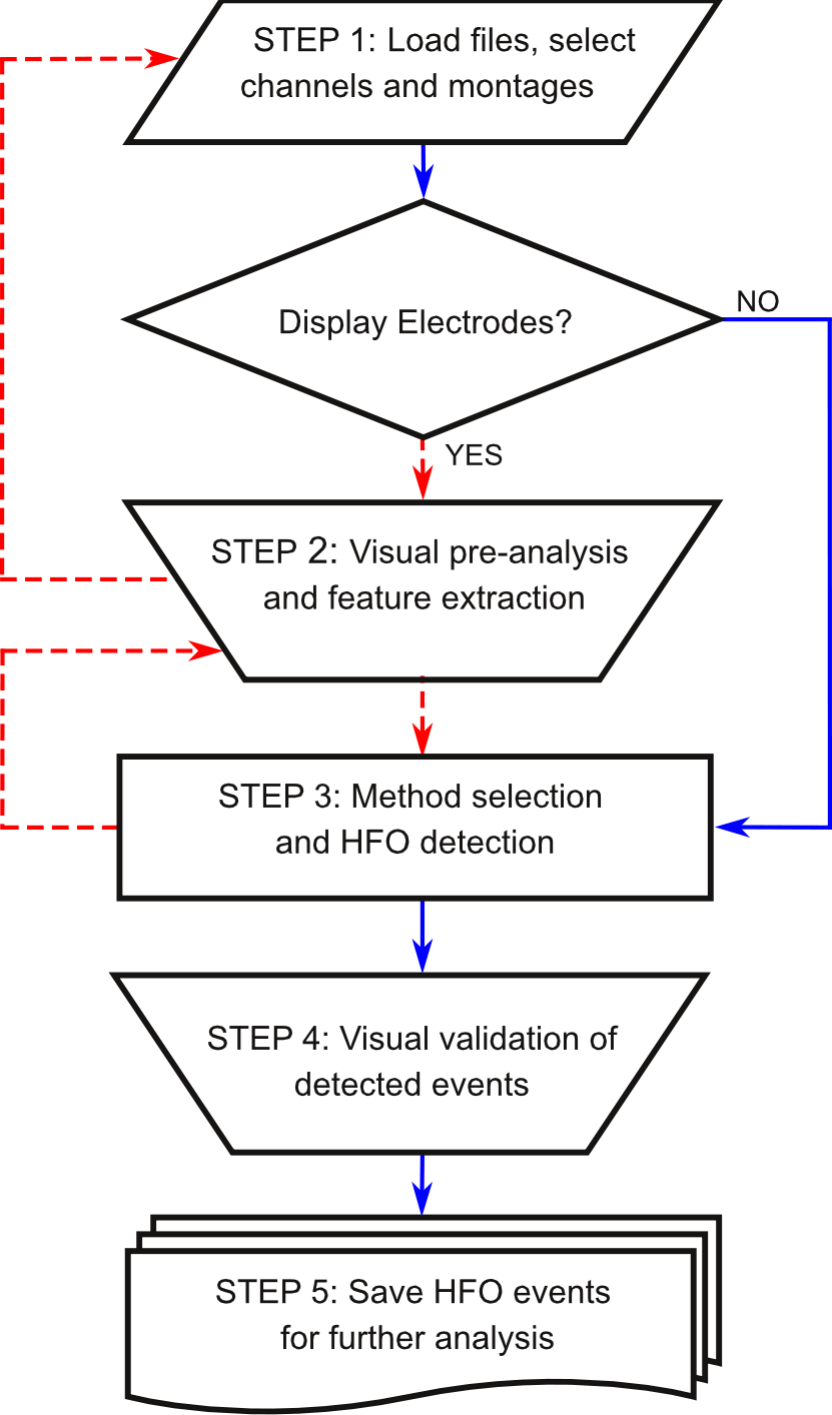
RIPPLELAB flowchart for data analysis. Visual pre-processing and feature extraction from displayed electrodes is an optional step but permits a better selection criterion before launching the automatic HFO detection. Red dashed lines connect blocks when electrodes are displayed.

The complete procedure for detection and analysis of HFOs through RIPPLELAB consists of several steps that are briefly presented in the following subsections and are summarized in **Fig 6**. They include different options for the general display and pre-processing of electroencephalographic data. Specific information about how to run the software is given in detail in the RIPPLELAB’s user manual, which is distributed along with the source code.

#### Step 1: Importing files and selecting channels

RIPPLELAB can import several file types including European Data Format, (*.rec, *.edf), Nicolet files (*.eeg), Neuralynx files (*.ncs), EPILEPSIAE file format (*.data) [41], EEGLAB files (*.set), Plexon files (*.plx), Axon binary format (*.abf), Micromed files (*.trc) and custom MAT Files (*.mat). Furthermore, when other MAT files do not meet the requirements, these files can be converted through RIPPLELAB to the appropriate format in order to be loaded into the system. Also, due to its modular structure, users and developers can easily add other file types if needed.

The user can simultaneously analyze one or several files of long-term recordings. Moreover, users can create and save custom bipolar or average montages for both display and analysis. Once the channel selection is done, the user can either visualize the data for pre-processing or go directly to implement the HFO detection. If this last option is chosen, RIPPLELAB does not load the entire file in memory; it only keeps the channel information for subsequent analysis. It is important to note that the software is set to load all the selected data into memory for further processing by default. Hence, when working with large files it is recommended to select only specific time segments of the focus channels to avoid overloading the system memory. For this, the user can modify the start and end times of the signal to be loaded.

#### Step 2: Displaying and pre-processing data

**Fig 7:**
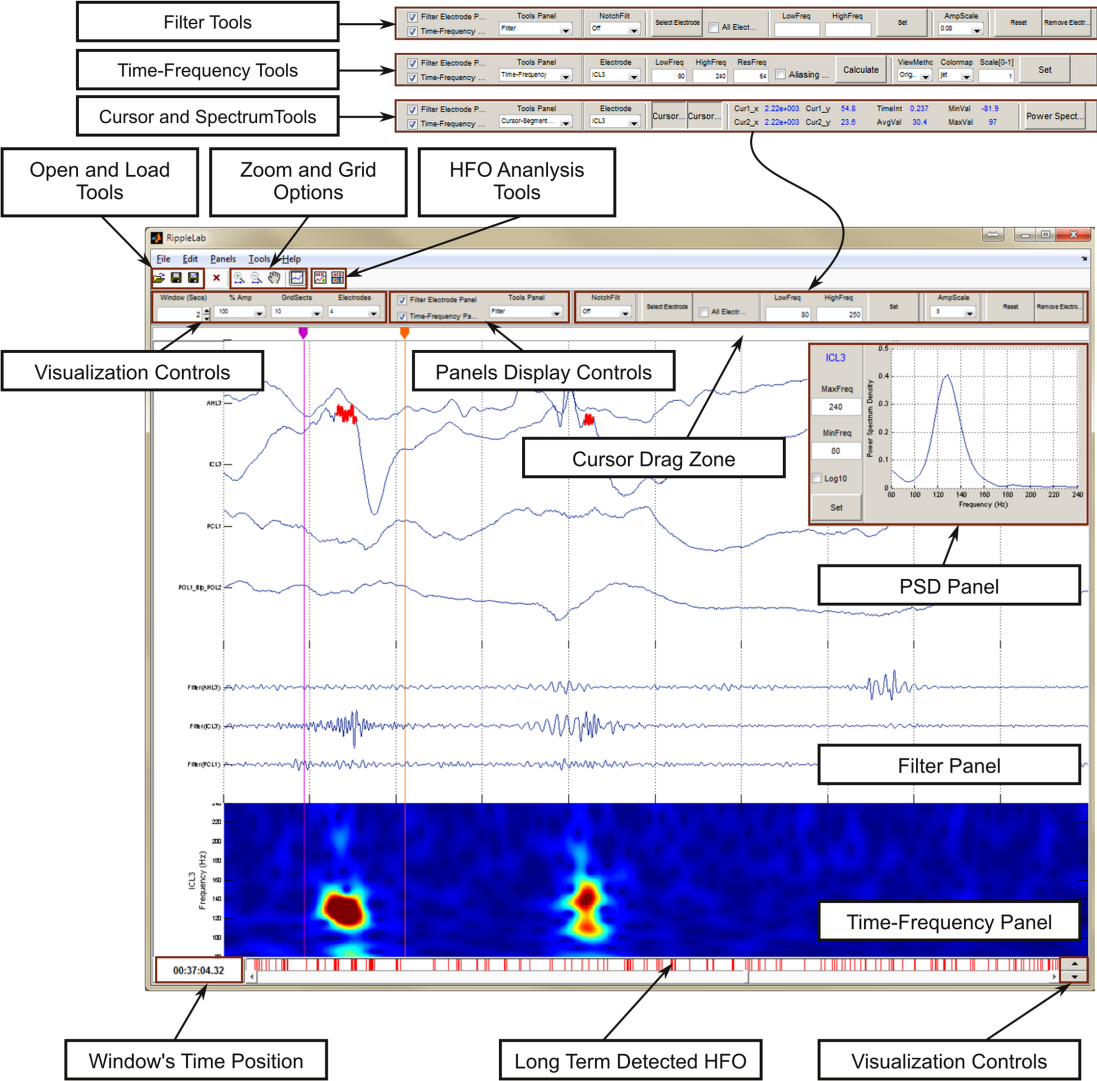
Features in RIPPLELAB main GUI. An intuitive environment for navigation has been developed, where the user can easily manipulate different options for the display of electrodes. Several options for signal analysis are supplied. These options give users the possibility to make better decisions on the selection of detection parameters. *Filter* and *Time-Frequency* panels can be shown or hidden as needed through the display control panel (*Panels Display Controls* box). Post visualization allows an easy navigation between detected events by presenting red vertical lines at each detected position.

As shown in **Fig 7**, RIPPLELAB offers useful options for visualization and pre-processing signals. This tool allows the user to visually explore the raw data and to compute different measures to better tune the HFO detection algorithms. This step is optional, and it is not required for HFO analysis.

The pre-processing options include filtering, time-frequency and spectral tools.

*1 –Filtering*: Different filter options can be implemented in RIPPLELAB. The user has the possibility to select between causal and non-causal filters to implement low-pass, high-pass and band-pass configurations with custom frequencies to the entire group of electrodes or to a selected group of channels. Likewise, a notch filter can be implemented at 50 or 60Hz with and without harmonics.

The *causal filter* implements a Hamming-windowed FIR digital filter of 50th order and a cutoff attenuation specified at -6dB [42]. The *non-causal filter* employs a type-II Chebyshev IIR forward-backward digital filter [43], which has a passband ripple of no more than 1 dB and a stopband attenuation of at least 20 dB, a cutoff attenuation specified at -3dB [42], and a second-order section implementation to maintain stability [44,45]. These filter characteristics allow the user to implement highly selective filters with narrow pass bands.

*2—Time-Frequency analysis*: The user has the possibility to perform a time-frequency transform of one selected electrode in a desired range of frequencies for the displayed interval. This time-frequency transform is estimated by the scalogram, which is computed with the continuous Gabor Wavelet [39,46] and is defined by the equations:

$$C\left(s,\tau \right)=\frac{1}{\sqrt{s}}\int_{-\infty }^{\infty }x\left(t\right)*\psi \left(\frac{t-\tau }{s}\right)dt$$ (3)

$$\psi \left(t\right)=\frac{1}{{\left(\sigma^{2}\pi \right)}^{1/4}}\exp\left(\frac{-t^{2}}{2\sigma^{2}}\right)e^{i\eta_{s}t}$$ (4)

where *t* indicates time, *s* represents scale, *τ* represents translation, *η*_*s*_ indicates the angular frequency at scale *s* and *σ* indicates the standard deviation of the Gaussian window in time. We set *σ* = 6/*η*_*s*_ in order to satisfy the admissibility condition [47,48].

*3—Cursors and power spectrum*: The user can estimate different measures such as time position, amplitude, time duration, and minimum, maximum and average amplitude of the segment between both cursors. Moreover, the power spectrum density (PSD) using the Welch estimation [49] can be obtained in a new overlapping panel for the segment between the cursors.

#### Step 3: HFOs detection methods

To provide a comprehensive support for HFO analysis, RIPPLELAB offers several alternatives for manual and automatic detection, visualization and manipulation of events. Each detection method includes a configuration panel, in which users can set the parameters and choose different analysis options.

#### Options for manual detection of HFO events

**Fig 8:**
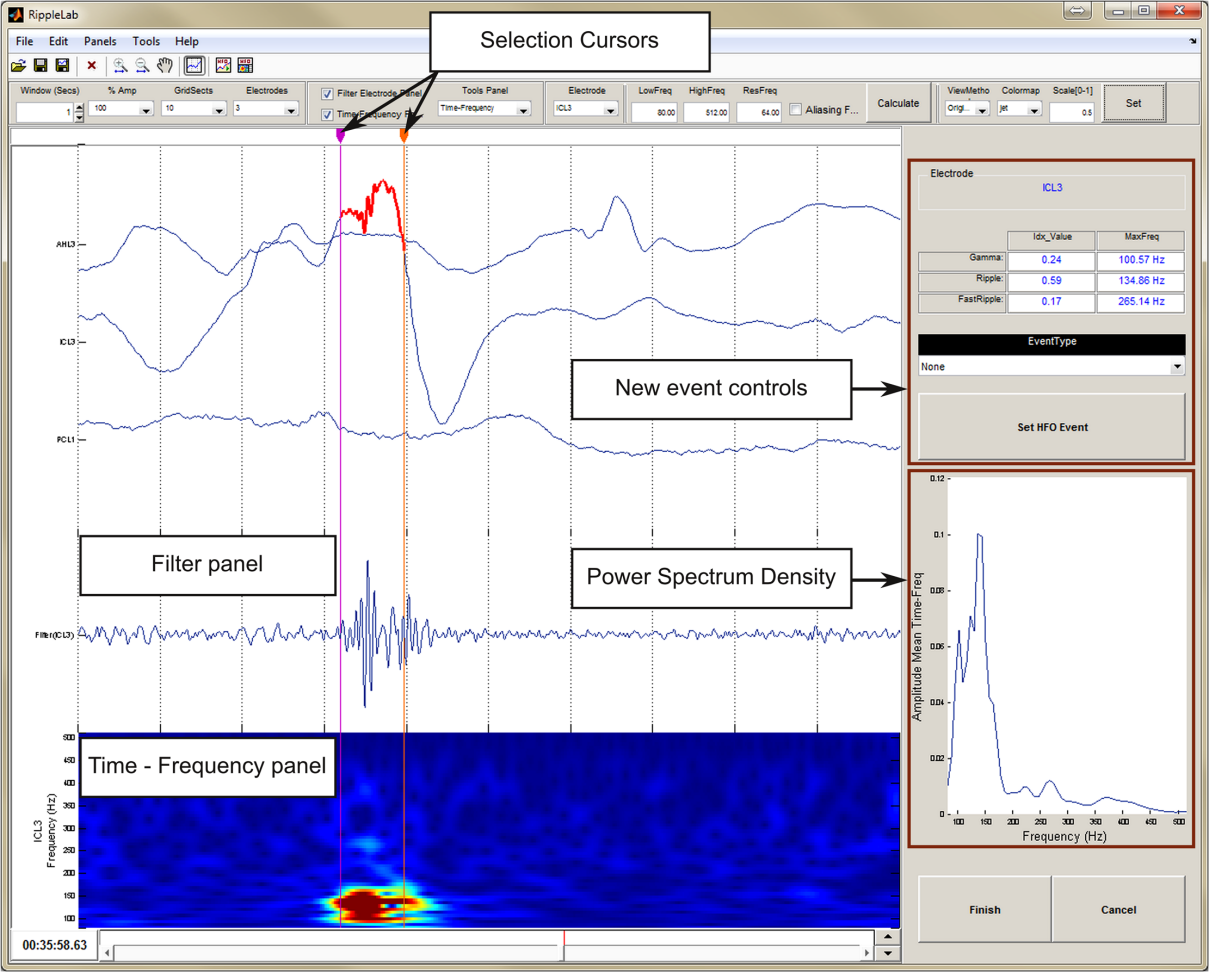
Visual Marking Panel. The visual marking panel allows the user the easy identification of HFOs through the visualization of time-frequency plot, filtered and PS of the displayed interval for a selected electrode. For the segment surrounded by cursors, the following measures are provided: PS, fast ripple index, ripple index and gamma index, including the maximum frequency for each band. Options for automatic detection of HFO events

The Visual Marking option assists the manual selection of HFO events depending on user criteria, and it can only be performed for a single electrode. This option displays the corresponding time-frequency plot together with the raw and filtered signals such as presented in **Fig 8**. In addition, the power spectrum of a selected segment is estimated and plotted. The user can classify selected events as gamma (40–120Hz), ripple (120–240Hz) or fast ripple (>240Hz).

**Fig 9:**
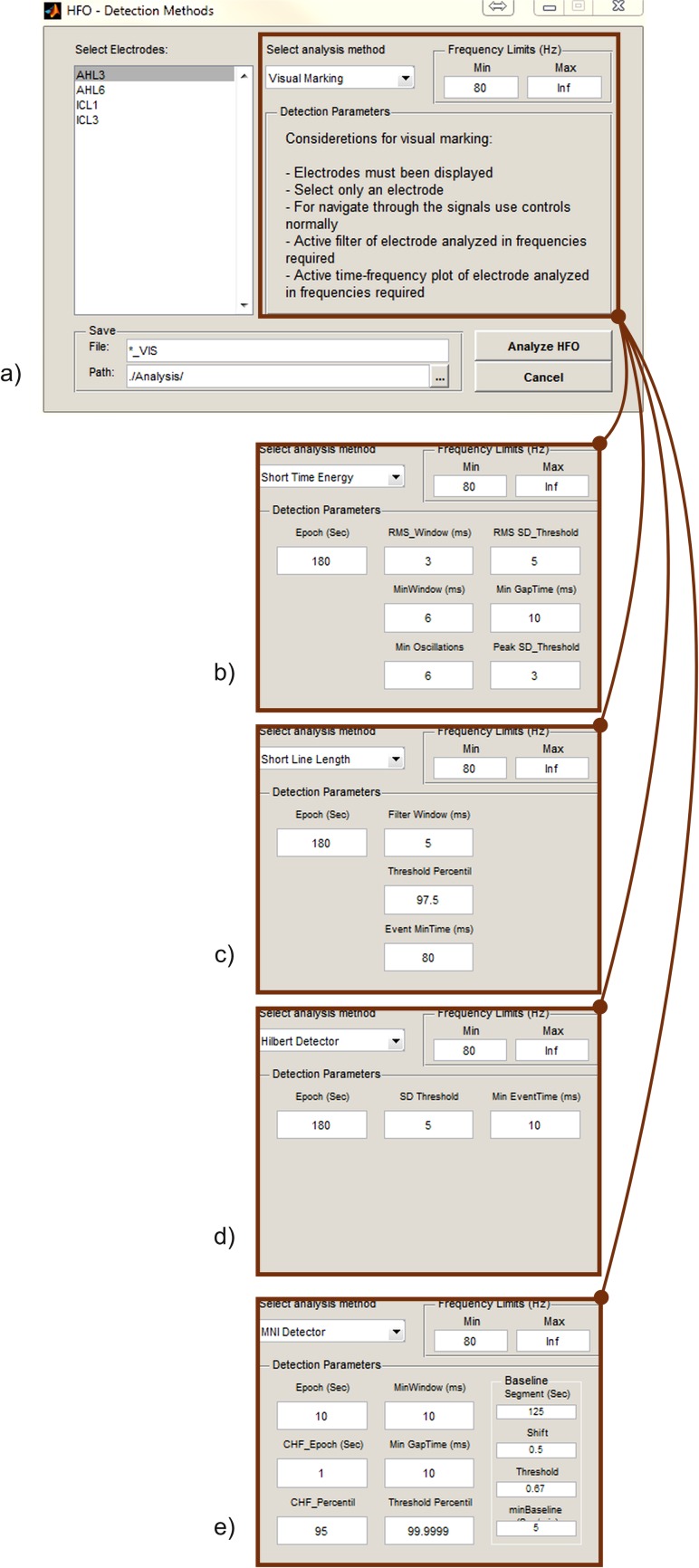
Detection Methods window. Besides selecting electrodes for detection, the user can select different parameters for the execution of the detection method. By default, these values correspond to those published in the respective original works. (a.) Visual marking. (b.) Short Time Energy Detector parameters. (c.) Short Line Length Detector parameters. (d.) Hilbert Detector parameters. (e.) MNI Detector parameters.

All of the implemented detection methods are configured by default with the parameters proposed in the original papers. However, the customization of these values is possible according to the user’s needs as displayed in **Fig 9**.

In order to help users to choose the appropriate method, some general considerations must be discussed. First of all, it is important to note that the methods we included in RIPPLELAB were designed to detect HFOs at different frequency bands. In fact, the STE detector was designed to find ripples in the frequency range between 80 and 175 Hz, and fast ripples between 200 and 600 Hz. The SLL method performs the detection over the frequency range > 80 Hz emphasizing high frequencies throughout the derivative filter. Distinctively, the HIL detector focuses on events at the frequency range between 180 and 400 Hz, and the MNI method centers its detection over the range from 80 to 450 Hz. Another difference across methods is their definition of event length. Specifically, the STE and MNI detectors state 6 ms and 10 ms, respectively, as event minimum lengths. A comparison of the implemented methods with different parameters was already performed in [14], where it was indicated that each of the detections methods can be optimized according to the database and the HFO characteristics that the researcher requires. The problem of selecting the optimal parameters is not trivial, and it has not been solved yet [9]

When selecting a method to implement, we first suggest setting the *Frequency Limits* parameter to the range of interest, and subsequently carry out the detection with all methods over a short-time interval of a well-known signal (e.g. 1-min EEG segment). It is recommended to test several threshold levels and epoch times. For instance, when increasing the threshold of a determined method, the sensitivity decreases, but the specificity rises [13,14]. Similarly, depending on the epoch time used for thresholding, the estimation of the local background energy differs due to changes in the vigilance state, artifacts and epileptic activity [34]. Usual epoch times for energy thresholding are 10-min, 5-min, 3-min and 1-min [10,31], though still, the user can establish a custom epoch time.

#### Inclusion of a new detection method in RIPPLELAB

RIPPLELAB allows advanced users to include new detection methods. For this, the scripts are written in MATLAB sections named according to the functionality of the code, and the object handles are stored in MATLAB structures. To facilitate the process of edition, the sections to modify when a new method is included are marked with the comment: [**INSERT!**], and an example is provided for each case. In general, to include a new detection method the following two features must be set: the visualization of the panel that allows the configuration of different parameters associated with the new method and the selection of the new method for further processing. Additional detailed information on the inclusion of a new detection method is provided in the user manual.

#### Step 4: HFOs visual validation

**Fig 10:**
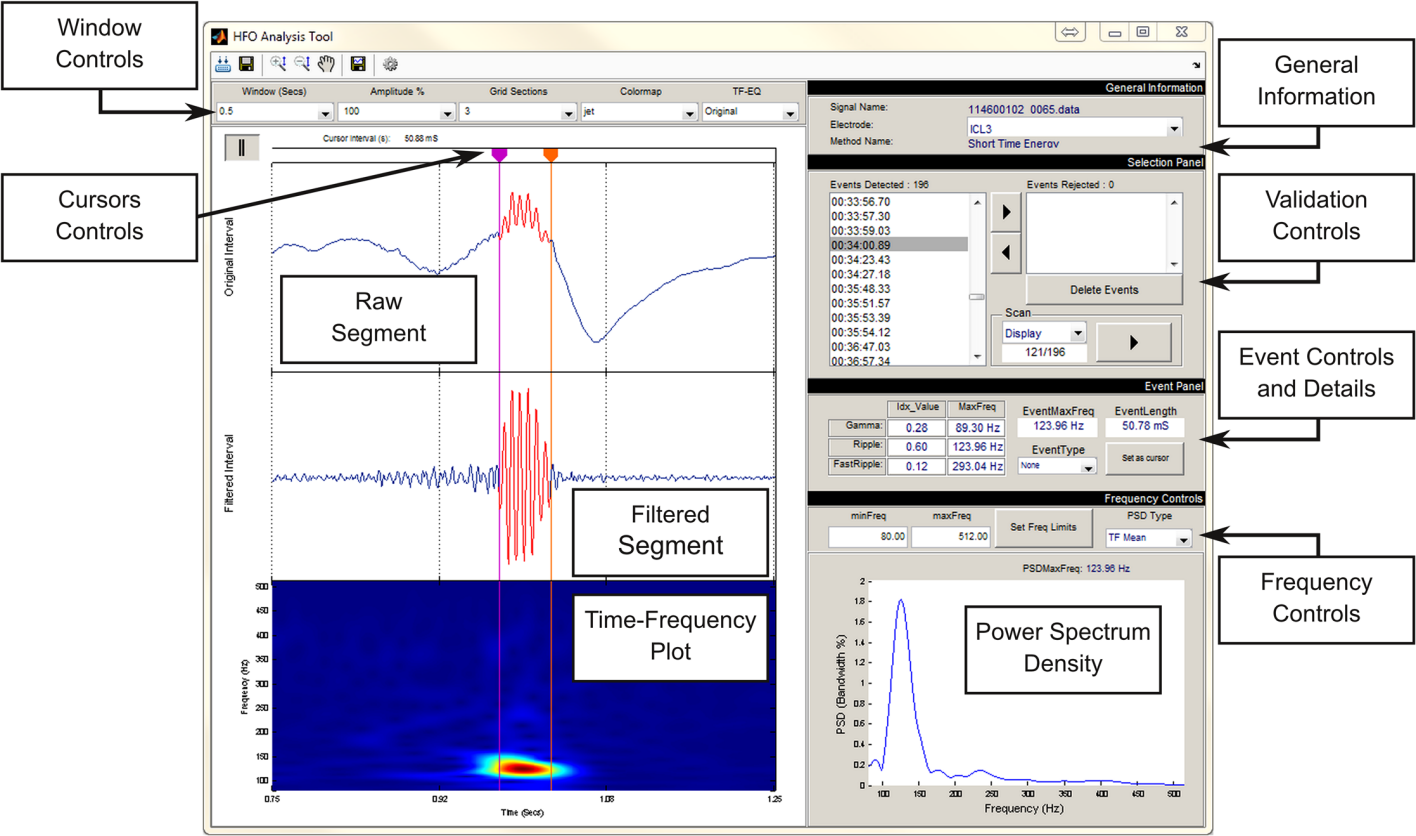
HFO Analysis Tool window for validation of detected HFOs. The electrode can be selected in *General Information* section. Navigation and deleting along detected events can be done in *Validation Controls*. *Frequency Controls* and *Window Controls* allow users to manage the visualization of axes displayed and *Power Spectrum Density*. *Event Controls* gives the possibility to re-locate the detected even or classify it as gamma, ripple, fast ripple or other type.

For a more detailed analysis of the detected HFOs, a special interface has been created (*HFO Analysis Tool*) to simplify the visual validation procedure performed by the user. This last GUI is presented in **Fig 10**, and it is composed of the following sections:

- The *General Information* panel presents the name of the analyzed file, the selected method for detection and the currently selected channel.
- The left panel presents a short segment of individual events. In the top panel, the raw signal is plotted highlighting the HFO in red. The equivalent filtered signal and the time-frequency plot are presented in middle and bottom panels, respectively.
- The *Selection Panel* allows users to visualize event by event. Valid HFOs can be confirmed, and spurious events can be rejected.
- The *Frequency Controls* panel allows the user to change the frequency limits for both the filtered signal and the time-frequency plot.
- The *Event* Panel allows the user to make manual adjustments to the temporal limits of the detected event through the time cursors provided for this effect. In this panel, the user can define the event type according to the classification: *Gamma*, *Ripple*, *Fast Ripple*, *Spike*, *Artifact or Other*. Additional types can be easily added if needed. Information about the maximum peak frequency and the time duration of the event is also provided. Lastly, the *Fast Ripple Index*, *the Ripple Index and the Gamma Index* are provided for reference [50], as well as the peak frequency of each band.

#### Step 5: Logging, saving and retrieving

**Fig 11:**
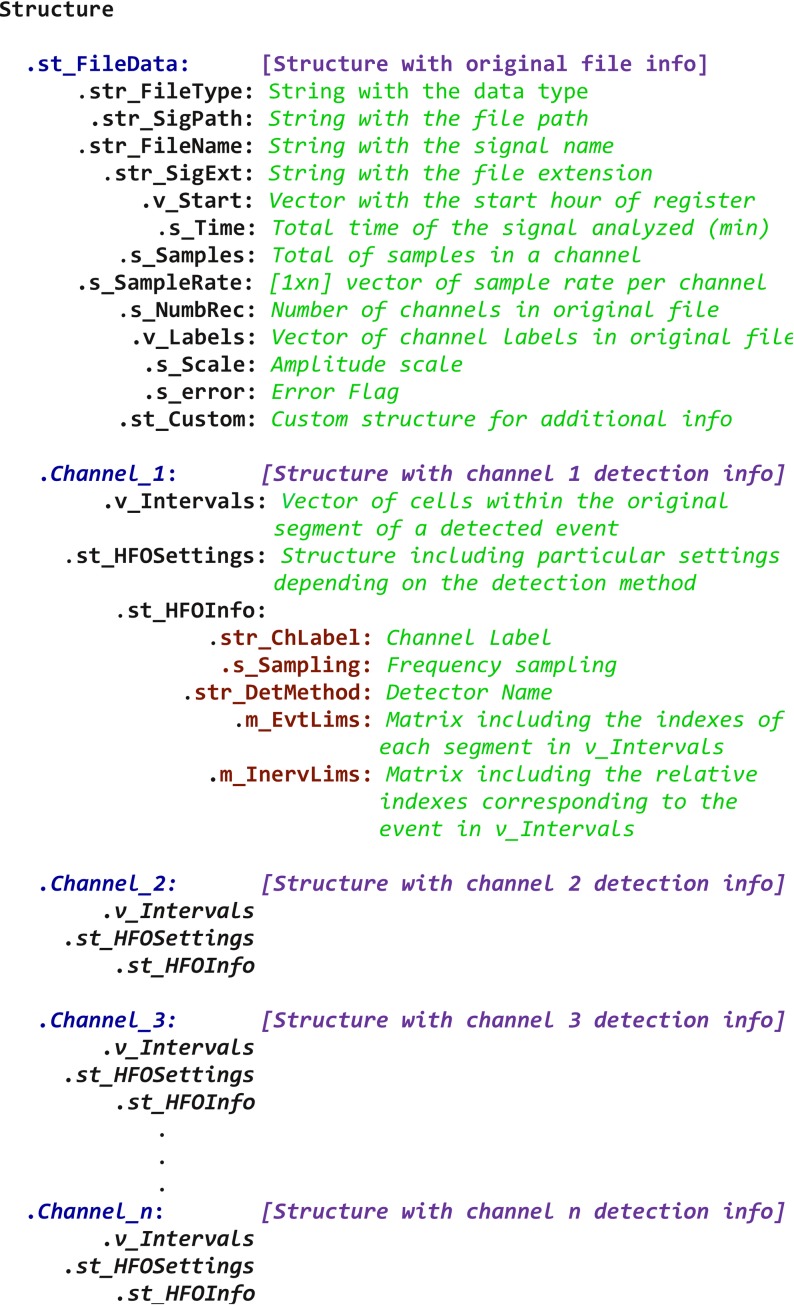
Structure proposed for HFO sharing. *Channel_n* names correspond to electrode labels of channels where putative electrodes were detected.

A main feature of RIPPLELAB is that the result of the analyses carried out during a session can be saved in files for future reviews, validations or sharing. For this purpose, RIPPLELAB proposes a custom-made structure MAT-File with the extension.*mat* modified to.*rhfe* which is created after each analysis. The general organization of this structure is described in **Fig 11**. Finally, logs of all operations accomplished during the detection procedure can be saved, which includes general information about the HFO detection method.

### Software validation

In order to minimize eventual programming errors in all developed scripts, we extensively and systematically tested each step of all the implemented detection algorithms using different types of data with distinct characteristics of noise, pathological activity and vigilance states. Extensive validations were also carried out on the different functionalities for signal preprocessing and inspection (display, filtering, spectral estimation, etc.). The performance and reliability of the tool for HFO analysis was also exhaustively evaluated for all detection methods on simulated and real data.

#### Simulated HFOs dataset

To test the HFO detection capabilities of RIPPLELAB, we first created a controlled scenario where the following types of events were simulated: spikes, gamma, ripple and fast ripple as well as different types of artifacts including 50 Hz noise. All events were implemented as sinusoids and Gaussian curves [33]. Gamma, ripples, and fast ripples were reproduced by implementing a sine wave of 125Hz, 225Hz and 325Hz respectively, and they were masked by a 50-percent cosine tapered window [51] in order to obtain a sine train with 8-oscillations within full amplitude. Spikes were reproduced using a Gaussian curve with a temporal duration of 30 ms containing ±3.7 SD. Artifacts were constructed using a discontinuity, and the 50Hz noise comprised harmonics in the 100–500 Hz range with a decreasing power-law amplitude. Both artifacts and noise were also masked by a 50-percent cosine tapered window to avoid discontinuities at the edges. To perform the detection of HFO events, the simulated activities were placed on top of a 30-min background signal of 1024 Hz sampling rate.

Two background cases were studied: non-background and real background. A zero-level signal was implemented in the non-background case, and a background activity from an intracranial EEG (iEEG) recording was used for the real background analysis. This electrophysiological interval was carefully chosen so that no high frequency events were present in the selected interval, which was visually confirmed by two experts. Similarly, the amplitude of this interval was normalized to have zero mean and one SD. In order to have a reasonable event amplitude fulfilling the spectral EEG power-law decay, we set the event amplitudes as next: Gamma = 3.5 SD, Ripple = 2.7 SD, Fast Ripple = 2.0 SD, Spike = 10 SD and artifacts and noise = 2 SD. Furthermore, spikes overlaid with fast ripples and spikes close to ripples (< 30 ms apart) were also simulated maintaining the same amplitude levels.

For both simulated background cases, all the detection methods were executed using the same parameters as published, and HFOs were detected for a frequency band between 80 and 500Hz. The sensibility and specificity of the detection was determined by the following equations:

$$Sensitivity=\frac{TP}{TP+FN}$$ (5)

$$Specificity=\frac{TN}{TN+FP}$$ (6)

where TP stands for true positives, TN for true negatives, FP for false positives and FN for False negatives. Detections of gamma, ripples, fast ripples and spike + fast ripples oscillations were considered as true detections, while spikes, artifacts and noise were considered as false detections.

#### Real EEG dataset

To assess RIPPLELAB’s capability to handle large databases––a common scenario found in clinic and research contexts––we completed an extensive analysis of 16 patients with invasive macro-electrodes from the EPILEPSIAE database [41]. This database contains long-term EEG recordings of epileptic patients (a total of 275) complemented with extensive metadata and standardized annotations of the data sets. These patients were all implanted with invasive macroelectrodes (depth electrodes, or subdural grids and strips) as a part of their clinical procedure to determine the epileptogenic zone. In our work, we first evaluated the automatic detection of HFOs from large amounts of data analyzing long-term EEG records, and then we implemented a visual validation for a group of candidate events.

For the first part of the analysis, two nights per patient were selected, one of them without any clinically marked seizure. For two patients, however, only one night was included because they did not present seizure-free nights. Only channels in the seizure onset zone were analyzed (n = 205 from 16 patients). A total of 3471 hours of iEEG sampled at 1024 Hz were processed using the STE and SLL automated detection methods. We defined epochs of 3-min for energy computation and a frequency range from 80 to 500 Hz for both of them.

For the second part of this analysis, we evaluated the type of oscillations that were identified for the implemented detection methods. To do this, 20-second channel segments with a high rate of detections, 100 to 1000 per hour, were randomly chosen from the results obtained in the previous analysis (n = 22). For these segments, the four automatic detection methods were applied and a visual validation procedure was performed. As suggested by Worrell et al. [4] and Menendez de la Prida et al. [9], only events with at least four oscillations and minimum duration of 25 ms between adjacent events were considered as HFOs. Events containing high and sharp amplitudes without a clear superimposed HFO were classified as Spikes [33]. The remaining events were categorized as noise or artifacts, and thus labeled as Other.

## Results

### Simulated data analysis

**Fig 12:**
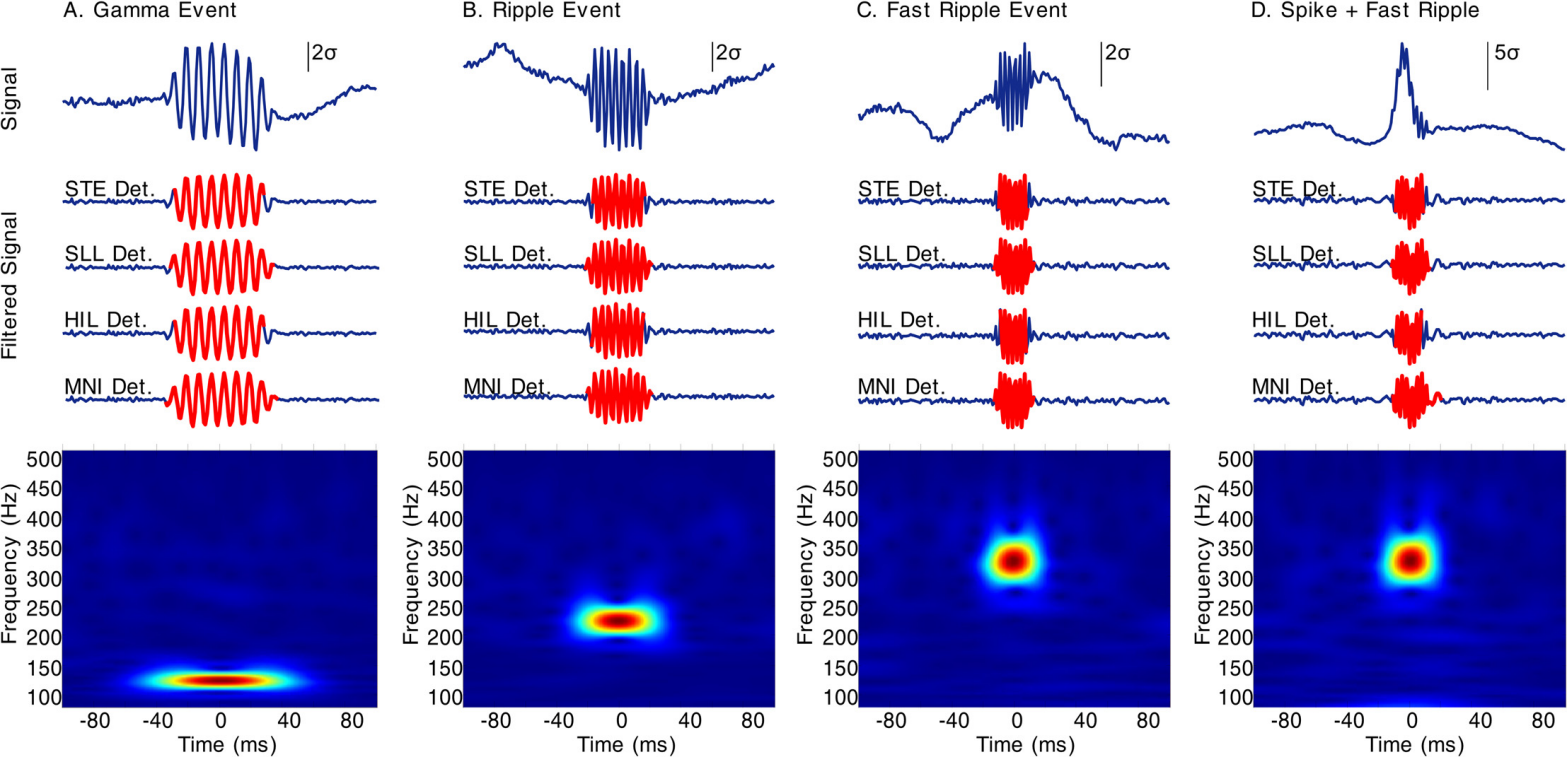
Simulated events over normalized real iEEG background. (A) Gamma, (B) Ripple, (C) Fast Ripple and (D) Spike + Fast Ripple events. TOP: Raw signal with the superimposed simulated event. MIDDLE: Filtered signal in the 80–500 Hz frequency band with the detected event for each method in red. BOTTOM: Time-Frequency plot for the raw signal segment. STE Det. (STE detection), SLL Det. (SLL detection), HIL Det. (HIL detection), MNI Det. (MNI detection).

For both background cases all methods accomplished high sensibility and specificity. Specifically, for the non-background case, all methods accomplished a sensibility and specificity of 100%. In addition, the event detection was consistent among each detection method, meaning that the event boundaries established for each method were uniform across all the events of the same type. It is important to note that for the MNI detector, the analysis was carried out using the detection for channels with baseline (right branch in **Fig 4**), which is expected because there are not components inside the band of interest besides the simulated events. In the real background case, sensitivities for STE, SLL and MNI detectors were 100%, and 99.33% for the HIL method. For this same case, the specificities were 100% for STE and HIL methods, 99.97% for SLL and 97.79% for the MNI detector. Nevertheless, event detection was also consistent among the implemented methods as can be seen in **Fig 12**. As expected, these results reveal that HFO detection is highly affected by the signal background. The results are also consistent with the specifications for SLL and MNI detectors which lose specificity in order to increase their sensibilities [13,14].

### Real data analysis

As a result of the first part of this analysis, the SLL method detected the largest number of events in comparison to the STE (1.040.437 and 252.642 respectively). On average, the STE method spent 32.07 ± 10.6 seconds analyzing hour-long segments whereas the SLL method performed slightly faster processing one hour segments in 30.52 ± 10.2 seconds. Nevertheless, the proportions of detected events using both methods were comparable for all but three patients. This result suggests that STE and SLL methods identify similar HFO densities on most of the signals even though the characteristics they use to evaluate HFOs are different, which is expected for different methods of high sensitivity. For a complete summary of the results, see **Table 1**.

**Table 1:** Performance comparison between STE and SLL methods over a large database.

Patient	Number of Electrodes	Time analyzed (Hours)	STE			SLL		
			Events detected	Processing time (Sec) (mean ± SD)	Event Detection Proportion	Events detected	Processing time (Sec) (mean ± SD)	Event Detection Proportion
pat_1	5	102,87	10.169	33.10 ± 6.1	4,03%	51.524	33.88 ± 10.7	4,95%
pat_2	8	172,29	3.071	33.18 ± 5.9	1,22%	14.922	35.34 ± 12.2	1,43%
pat_3	40	365,53	38.550	31.76 ± 9.5	15,26%	73.439	26.10 ± 7.1	7,06%
pat_4	20	429,87	30.560	34.27 ± 4.1	12,10%	140.863	32.12 ± 3.0	13,54%
pat_5	6	128,27	5.368	34.59 ± 11.7	2,12%	13.384	34.44 ± 11.8	1,29%
pat_6	11	220,18	14.548	32.26 ± 4.6	5,76%	55.301	32.26 ± 4.5	5,32%
pat_7	5	100,00	3.081	32.62 ± 3.0	1,22%	28.822	34.20 ± 12.0	2,77%
pat_8	7	63,00	3.174	34.45 ± 12.2	1,26%	25.025	31.42 ± 5.5	2,41%
pat_9	7	136,10	33	7.98 ± 1.5	0,01%	151.028	6.67 ± 0.7	14,52%
pat_10	16	305,27	27.448	35.47 ± 19.9	10,86%	176.545	34.60 ± 19.0	16,97%
pat_11	5	99,66	3.527	32.01 ± 13.3	1,40%	40.403	33.46 ± 11.5	3,88%
pat_12	22	232,78	5.448	32.62 ± 8.4	2,16%	36.737	30.80 ± 7.2	3,53%
pat_13	4	76,79	3.075	29.76 ± 10.6	1,22%	12.722	28.45 ± 7.7	1,22%
pat_14	9	192,49	16.637	32.64 ± 4.1	6,59%	74.320	32.58 ± 3.6	7,14%
pat_15	19	389,80	13.150	30.96 ± 7.1	5,20%	62.471	29.20 ± 6.4	6,00%
pat_16	21	456,38	74.803	36.13 ± 3.1	29,61%	82.931	31.56 ± 2.0	7,97%
**TOTAL**	205	3.471,27	252.642	-	-	1.040.437	-	-
**AVERAGE**	12,81	216,95	15.790,13	32.07 ± 10.6	-	65.027,31	30.52 ± 10.2	-

**Fig 13:**
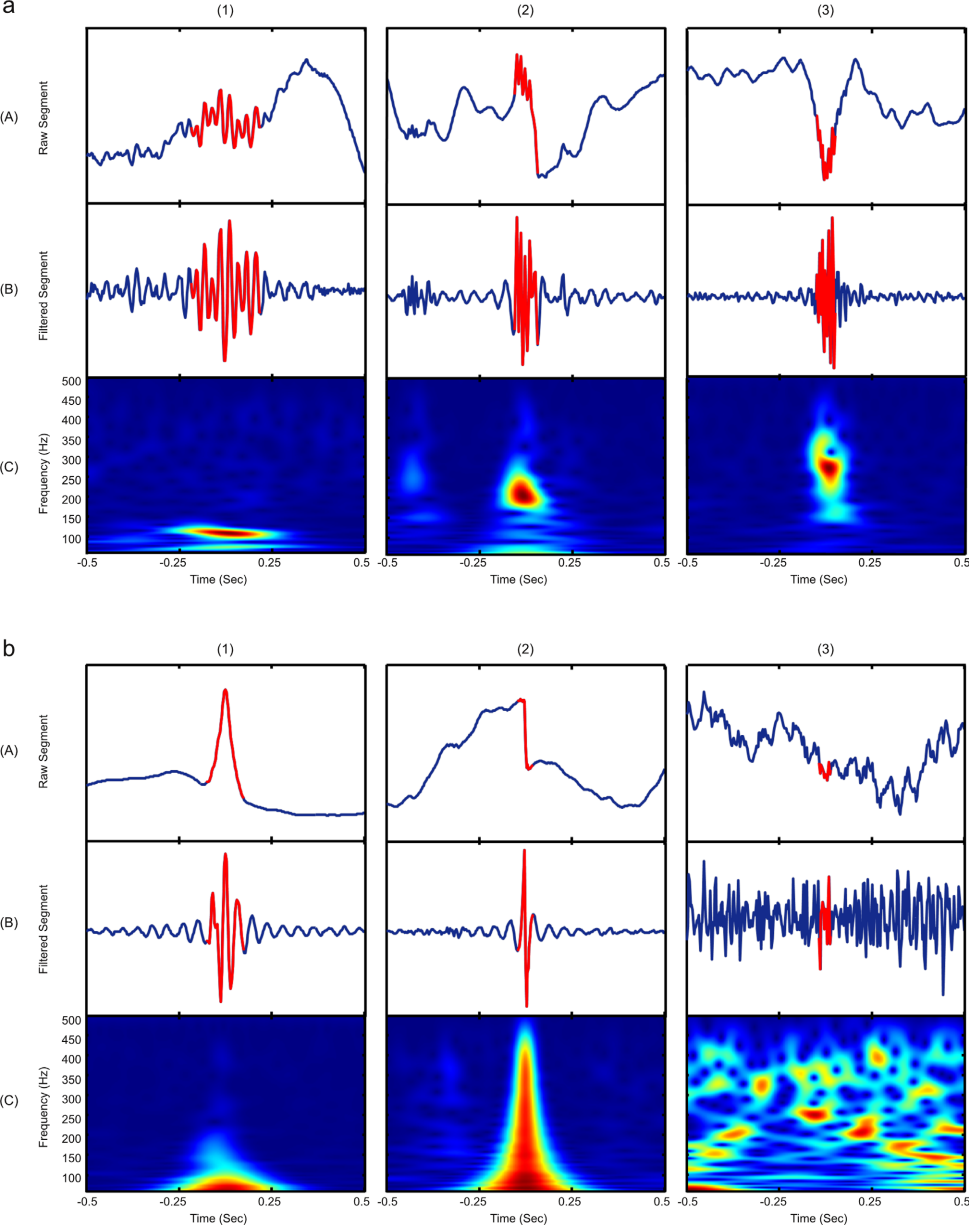
Typical events found during the evaluation procedure are presented over a 300 ms window. The filter and time frequency scalogram has been established in the frequency band from 60 Hz to 500 Hz. Examples of (a) correct HFO detections and (b) false detections are displayed. As shown in the *HFO Analysis Tool* interface, figures in rows (A) correspond to raw data from detected events, filters of such events are presented in row (B) and time frequency plots in row (C). For detected events, figure (a1) shows a gamma event, (a2) a ripple and (a3) a fast ripple. For false detections, figure (b1) presents a detected spike, (b2) an artifact and (b3) an example of background noise.

For the second part of the analysis, a total of 14.804 events were visually reviewed for an expert. For all methods, 4542 events were classified as valid HFOs, 5115 as spikes, and 4967 as others. In our study, the MNI detector presented the highest number of HFO detections (n = 1929), but also had the highest level of false detections (true HFOs events represented only 27% of total detections). Conversely, the STE detector presented the lowest number of HFOs detected events but with the highest true detection rate (n = 418 corresponding to 43% of total detections). Moreover, the HIL method obtained the highest spike detection rate (50%) and SLL the highest noise detection rate (49%). For a complete summary of the results see **Table 2**. Examples of different types of detected events are present in **Fig 13**.

**Table 2:** Event classification for each HFO detection method after visual validation of selected segments.

*Patient*-Segment	STE			TOTAL	SLL			TOTAL	HIL			TOTAL	MNI			TOTAL
	Other	Spikes	HFOs		Other	Spikes	HFOs		Other	Spikes	HFOs		Other	Spikes	HFOs	
*pat_1–*1	47	0	0	47	331	2	27	360	100	11	1	112	363	35	21	419
*pat_1–*2	0	22	43	65	6	33	56	95	3	94	30	127	17	192	139	348
*pat_2–*1	1	0	82	83	16	8	223	247	4	25	198	227	44	82	336	462
*pat_3–*1	0	2	71	73	4	8	110	122	4	22	142	168	7	22	194	223
*pat_4–*1	4	13	1	18	30	29	5	64	12	57	14	83	131	88	32	251
*pat_4–*2	19	24	1	44	118	34	0	152	69	79	6	154	226	164	11	401
*pat_5–*1	0	42	0	42	36	34	2	72	2	73	3	78	23	105	7	135
*pat_5–*2	0	15	20	35	22	25	52	99	2	58	44	104	9	208	67	284
*pat_6–*1	1	62	0	63	90	29	0	119	0	153	0	153	68	205	4	277
*pat_6–*2	3	6	0	9	16	0	0	16	3	12	0	15	34	15	0	49
*pat_7–*1	4	39	0	43	149	15	14	178	6	79	0	85	97	95	0	192
*pat_8–*1	14	3	50	67	90	39	304	433	49	30	37	116	174	84	180	438
*pat_10–*1	4	34	6	44	121	67	22	210	14	110	18	142	102	187	23	312
*pat_10–*2	16	31	15	62	88	43	64	195	24	85	22	131	161	116	95	372
*pat_12–*1	1	1	47	49	49	0	158	207	4	6	94	104	17	6	135	158
*pat_13–*1	10	0	0	10	114	0	0	114	10	0	0	10	151	0	0	151
*pat_14–*1	37	0	0	37	210	0	0	210	66	0	0	66	472	0	0	472
*pat_14–*2	42	0	0	42	102	0	0	102	84	0	0	84	214	0	0	214
*pat_15–*1	2	27	0	29	112	137	0	249	3	317	0	320	60	645	0	705
*pat_15–*2	0	39	0	39	1	132	42	175	0	246	101	347	0	439	157	596
*pat_16–*1	0	0	30	30	43	5	28	76	7	48	165	220	99	62	275	436
*pat_16–*2	0	0	52	52	3	5	57	65	31	4	156	191	45	31	253	329
**TOTAL DETECTIONS**	**205**	**360**	**418**	***983***	**1751**	**645**	**1164**	***3560***	**497**	**1509**	**1031**	***3037***	**2514**	**2781**	**1929**	***7224***
**PROPORTION (%)**	**21%**	**37%**	**43%**	**-**	**49%**	**18%**	**33%**	**-**	**16%**	**50%**	**34%**	**-**	**35%**	**38%**	**27%**	**-**

Although the main purpose of our analyses was to evaluate RIPPLELAB’s capabilities and not to perform a rigorous comparison between methods, our results are consistent with a previous study that made a comparison of these four algorithms [14]. In particular for that study, using the default parameters, the STE method achieved a sensitivity of 38.1% and a specificity of 100%, the SLL method achieved sensitivity of 27.6% and a specificity of 92%, the HIL detector achieved sensitivity of 21.1% and a specificity of 90% and the MNI detector achieved sensitivity of 91% and a specificity of 91%. Thus, in concordance with our own results, highest sensitivity and specificity were obtained with the MNI detector presented higher sensitivity and the STE detector presented higher specificity respectively. Altogether, these results demonstrate that RIPPLELAB succeeds in reducing the analysis complexity for the HFO reviewer, and that this application properly identifies putative HFOs. All the above indicates that RIPPLELAB is a valuable tool for HFO analysis.

## Discussion

HFO detection and analysis is a difficult task that requires the use of different tools such as detection algorithms, diverse signal processing techniques and specific visualization options. Even though several computational tools exist for the analysis of neural data, none of them includes the appropriate graphical environment nor the implementation of any detection method for HFO analysis, for which our software tool is particularly oriented. For this reason RIPPLELAB becomes a unique tool that consolidates a variety of current analytic methodologies for the analysis of these type of events.

RIPPLELAB is a free-of-charge and open source software tool that includes both manual and automatic detection of HFO events. It can handle different types of electrophysiological data such as invasive and scalp EEGs through several file formats. The automatic detection incorporates four already-published methods. Furthermore, RIPPLELAB provides a GUI to facilitate the visual validation of detected events. Especially noteworthy are the possibility to automatically analyze large files, and the possibility to save all the analyses. The resulting files can be further reviewed and easily shared by different groups to be compared conveniently. The RIPPLELAB code was developed in a modular manner, making possible the integration of new methods for HFO detection and the continuous development of new characteristics. The RIPPLELAB capabilities were tested through the analysis of simulated and real signals from iEEG recordings of epileptic patients. These results were consistent with previous studies where the detection algorithms were studied and compared. This fact attest to the reliable operation of RIPPLELAB as a tool for HFO analysis.

RIPPLELAB was developed for users of different technical backgrounds, so it provides access to powerful methods for HFO detection without the necessity for detailed knowledge of methodological aspects. Due to its characteristics, RIPPLELAB could serve as a standard platform for testing and comparing new or existing HFO detection and analysis methodologies. Because of the relevance of HFOs in epilepsy, we think that this tool will be particularly useful in clinical and research contexts associated with this pathology, and we hope that this tool could promote and simplify the collaboration and exchange of information between centers working in the field of HFOs.
